# AGEIST ATTITUDES AND PREPARATION AS INDIRECT PREDICTORS OF TRAINEES’ INTEREST IN CLINICAL WORK WITH OLDER ADULTS

**DOI:** 10.1093/geroni/igae098.3098

**Published:** 2024-12-31

**Authors:** Grace Caskie, Mackenzie Kirby, Eve Root

**Affiliations:** Lehigh University, Bethlehem, Pennsylvania, United States; Lehigh University, Bethlehem, Pennsylvania, United States; Lehigh University, Clayton, Missouri, United States

## Abstract

The striking discrepancy between the number of older adults in need of mental healthcare and the small number of psychologists with specialized training in providing this care (Moye et al., 2019) underscores the importance of identifying modifiable factors that could increase psychology trainees’ interest in future clinical work with older adults. We tested a structural equation model specifying ageist attitudes and preparation regarding aging as having indirect effects on interest in working with older adults through three measures of perceived work with older adults. Doctoral trainees (N=187; age=22-43 years) in clinical/counseling psychology completed the Fraboni Scale of Ageism, a 10-point item assessing trainees’ perceptions of how well their program prepared them to work with older adults, the Working with Older Adults Scale (assessing perceptions of ease/difficulty of work, social approval for work, favorable evaluation of work, and interest in work), the Social Desirability Scale, and demographic items. Controlling for trainees’ age and social desirability tendencies, the model demonstrated good fit (CFI=.95, TLI=.94, RMSEA=.07). Significant indirect effects were found for ageist attitudes (p<.001) and preparation (p=.01) on interest. More ageist attitudes related significantly to greater perceived difficulty (p<.001), less favorable evaluations (p<.001), and less social approval (p=.02) regarding working with older adults, while greater preparation related only to less difficulty of working with older adults. Difficulty, social approval, and evaluation of work with older adults each significantly predicted interest (p<.001). Implementing training opportunities for trainees that aim to reduce ageist attitudes could increase interest in working with the older adult population.

